# Supplementary material for: A dynamical limit to evolutionary adaptation
Source: bioRxiv. 2023 Aug 2:2023.07.31.551320. Preprint. [Version 1] doi: 10.1101/2023.07.31.551320 (PMC10418092; doi:10.1101/2023.07.31.551320)
Supplement: 1 [file NIHPP2023.07.31.551320V1-supplement-1.pdf]

bioRxiv preprint doi: https://doi.org/10.1101/2023.07.31.551320; this version posted August 2, 2023. The copyright holder for this preprint (which was not certified by peer review) is the author/funder, who has granted bioRxiv a license to display the preprint in perpetuity. It is made available under aCC-BY 4.0 International license.

SI Appendix: A dynamical limit to evolutionary adaptation

Matthew J. Melissa and Michael M. Desai

(Dated: July 31, 2023)

I. REVIEW OF THE MODERATE SELECTION, STRONG MUTATION APPROXIMATION

In this section we provide a brief overview of the moderate selection, strong mutation (MSSM) approximation, which can be used to analyze evolutionary dynamics in the MSSM regime. This summarizes the key results of Melissa et al. (2022) that are relevant to our characterization of the $v = 0$ constraint.

A central aim is to predict, given the parameters N and $U\rho(s)$, the dynamical quantities v and $p_{\text{fix}}(s)$, along with the timescale T_c of pairwise coalescence. A key result of Melissa et al. (2022) is that under the MSSM approximation,

$$p_{\text{fix}}(s) \approx \frac{e^{T_c s}}{N}, \tag{1}$$

for $s \ll b$, where T_c is defined by

$$v \equiv U \int \rho(s) s e^{T_c s} ds, \tag{2}$$

and b is defined by

$$b^3 \equiv \frac{U}{2} \int \rho(s) s^2 e^{T_c s} ds. \tag{3}$$

Note that to apply the MSSM approximation, $\rho(s)$ must fall off exponentially, or faster than exponentially, with large positive s; otherwise, the above integrals do not converge and T_c is not well-defined. The quantity T_c is related to the underlying parameters N and $U\rho(s)$ through the following system of equations for T_c and the interference threshold x_c (the fitness advantage above which individuals can fix largely unhindered by clonal interference, as well as, roughly speaking, the typical fitness advantage of the most-fit individuals in the population):

$$1 - T_c x_c \left[1 - \frac{1}{T_c b} \frac{\text{Ai}'\left(\frac{c-x}{b}\right)}{\text{Ai}\left(\frac{c-x}{b}\right)}\right] = 0, \tag{4}$$

and

$$\left[\frac{\left(\text{Ai}'\left(\frac{c-x_c}{b}\right)\right)^2 - \left(\frac{c-x_c}{b}\right)\text{Ai}^2\left(\frac{c-x_c}{b}\right)}{\text{Ai}\left(\frac{c-x_c}{b}\right)}\right] N x_c = e^{T_c x_c - \frac{v T_c^2}{2} + U \int \frac{\rho(s) ds}{s}\left(e^{T_c s} - T_c s - 1\right)}, \tag{5}$$

where

$$c \equiv U \int \rho(s) \left[T_c s e^{T_c s} + 1 - e^{T_c s}\right] ds. \tag{6}$$

Eq. (4) and Eq. (5) are used throughout this work to obtain theory predictions under the MSSM approximation. While numerical solution of these equations is straightforward, it is sometimes useful to further approximate these two equations as

$$x_c \approx c - b z_0 - 1/T_c, \tag{7}$$

and

$$\log N x_c \approx T_c(x_c - U) - \frac{v T_c^2}{2} + U \int \frac{\rho(s) ds}{s}\left(e^{T_c s} - 1\right), \tag{8}$$

bioRxiv preprint doi: https://doi.org/10.1101/2023.07.31.551320; this version posted August 2, 2023. The copyright holder for this preprint (which was not certified by peer review) is the author/funder, who has granted bioRxiv a license to display the preprint in perpetuity. It is made available under aCC-BY 4.0 International license.

respectively, which holds in the regime of validity of the MSSM approximation (which we discuss below).

While defined by Eq. (2), the quantity T_c approximately corresponds to $\langle T_2 \rangle /2$—one-half the average time since two randomly chosen individuals in a population share a common ancestor—and can be interpreted as a *coalescence timescale*. In particular, the pairwise neutral heterozygosity π_{neu} (the average number of neutral genetic differences among pairs of individuals in a population) evaluates approximately to $4T_cU_n$; the relation $\langle T_2 \rangle /2 \approx T_c$ follows since $\pi_{\text{neu}} = 2\langle T_2 \rangle U_n$. The pairwise *selected* heterozygosity (which counts *selected* genetic differences, occurring at rate U, with fitness effects drawn from $\rho(s)$) is given by

$$\pi_{\text{sel}} = 2T_cU \int \rho(s) \left[e^{T_c s} + \left(\frac{e^{T_c s} - 1}{T_c s} \right) \right] ds. \tag{9}$$

Results have also been obtained for the full site frequency spectrum (which gives the expected number of mutations present in a sample at a given frequency, and from which additional statistics of genetic diversity can be computed) for both neutral mutations and selected mutations. The fixation rate of neutral mutations is simply given by U_n, while the fixation rate of selected mutations is given by $F = U \int \rho(s) e^{T_c s}$; from these prediction, a prediction for α (as defined in the main text) follows.

Approximate validity of the MSSM approximation requires that $T_c b \gg 1$ and that

$$\frac{\int \rho(s) e^{T_c s} \left(e^{s/b} - 1 - s/b - \frac{1}{2}(s/b)^2 \right) ds}{\frac{1}{2} \int \rho(s) e^{T_c s} (s/b)^2 \, ds} \ll 1. \tag{10}$$

For populations subject purely to beneficial mutaitons, or purely to deleterious mutations, the second condition is roughly encapsulated by the condition $\tilde{s} \ll b$, where $\tilde{s} \equiv \langle s_f \rangle + 2\Delta s_f$ denotes the largest "typical" effect size of a fixed mutation, as well as by the requirement that $\rho(s)$ falls off exponentially, or faster than exponentially with large positive s. The quantity $\langle s_f \rangle$ denotes the *average* effect size of a fixed mutation, and the quantity Δs_f denotes the *standard deviation* in effect sizes of fixed mutation. When beneficial and deleterious mutations both occur in a population, separate largest "typical" effects $\tilde{s}_b$ and $\tilde{s}_d$ can defined for beneficial and deleterious mutations, and both $\tilde{s}_b \ll b$ and $\tilde{s}_d \ll b$ must be satisfied. These conditions of validity must be satisfied self-consistently; provided that these conditions are met, the distribution $\rho_f(s)$ of *fixed* mutational effects follows as $\rho_f(s) \propto \rho(s) e^{T_c s}$ within the "bulk" of $\rho_f(s)$—the region dominating $\int \rho_f(s) ds$. These conditions ensure that the distribution of relative fitnesses $f(x)$ is well-approximated by

$$f(x) \propto e^{-T_c x} \text{Ai} \left(\frac{c - x}{b} \right), \tag{11}$$

and that the fixation probability $w(x)$ of an individual with relative fitness x is well-approximated by

$$w(x) \propto e^{T_c x} \text{Ai} \left(\frac{c - x}{b} \right), \tag{12}$$

within the important region of fitness space (the "fixation class", near the "nose" of the fitness distribution) which produces the majority of future common ancestors of the population. This region of fitness space (which dominates the integral $\int f(x) w(x) dx$) is particularly important because Eq. (8) is obtained by enforcing that $1/N = \int f(x) w(x) dx$ (i.e., that on average, one individual in the population at any given time will eventually fix).

II. APPLICABILITY OF THE MSSM APPROXIMATION ALONG THE $v = 0$ RIDGELINE

In the main text, we argued that the $v = 0$ ridgeline (in the space of scaled fitness effects) is given by the curve $\gamma_d^* = 1$ and $\gamma_b^* = W\left(\frac{1}{e\eta}\right)$, parameterized by η. Here, we evaluate the quantities $T_c b$, s_d/b and s_b/b along this $v = 0$ ridgeline, which we then use to comment on the validity of the MSSM approximation in describing the $v = 0$ ridgeline.

From the definition of b in Eq. (3), it follows that

$$(T_c b)^3 = \frac{T_c}{2} \left[U_b (\gamma_b^*)^2 e^{\gamma_b^*} + U_d (\gamma_d^*)^2 e^{-\gamma_d^*} \right] = \frac{T_c}{2} \left[\frac{U_b}{e\eta} \gamma_b^* + \frac{U_d}{e} \right] \tag{13}$$

bioRxiv preprint doi: https://doi.org/10.1101/2023.07.31.551320; this version posted August 2, 2023. The copyright holder for this preprint (which was not certified by peer review) is the author/funder, who has granted bioRxiv a license to display the preprint in perpetuity. It is made available under aCC-BY 4.0 International license.

on the $v = 0$ ridgeline, which in turn simplifies to

$$(T_c b)^3 = \frac{T_c U_b}{2e\eta}\left[1 + W\left(\frac{1}{e\eta}\right)\right]. \tag{14}$$

As a consequence, $T_c b \gg 1$ and $s_d/b \ll 1$ if $T_c U_b \gg 1$ (assuming $\eta < 1$). Furthermore, given Eq. (14) we have

$$\left(\frac{s_b}{b}\right)^3 = \frac{2e\eta W\left(\frac{1}{e\eta}\right)}{T_c U_b\left[1 + W\left(\frac{1}{e\eta}\right)\right]}, \tag{15}$$

with the right-hand side of Eq. (15) bounded above by $0.4/(T_c U_b)$; thus, $s_b/b \ll 1$ along the $v = 0$ ridgeline if $T_c U_b \gg 1$.

III. DISTRIBUTIONS OF FITNESS EFFECTS

Here, we consider simple classes of distributions of fitness effects, simplifying expressions obtained in the main text, and justifying the use of the MSSM approximation in describing the $v = 0$ ridgeline. We focus our attention on the case in which effect sizes of both beneficial mutations and deleterious mutations are drawn from gamma distributions, potentially differing in shape and scale. That is, we assume

$$U\rho(s) = \begin{cases} \frac{\alpha_d U_d}{\Gamma(\alpha_d)s_d}\left(\frac{\alpha_b|s|}{s_d}\right)^{\alpha_d - 1} e^{-\alpha_d|s|/s_d} & s < 0 \\ \frac{\alpha_b U_b}{\Gamma(\alpha_b)s_b}\left(\frac{\alpha_b s}{s_b}\right)^{\alpha_b - 1} e^{-\alpha_b s/s_b} & s > 0 \end{cases}, \tag{16}$$

with s_b and s_d the mean available effect size of a beneficial mutation, or a deleterious mutation, respectively—that is, $s_b \equiv \int \rho_b(s)sds$ and $s_d \equiv \int \rho_d(s)|s|ds$. The parameters α_b and α_d comprise the shape parameters of the respective gamma distributions; the choice $\alpha_b = 1$ (or $\alpha_d = 1$) corresponds to an exponential distribution of beneficial (or deleterious) fitness effects; the limit $\alpha_b \to \infty$ (or $\alpha_d \to \infty$) corresponds to a delta distribution of beneficial (or deleterious) fitness effects.

Given the $U\rho(s)$ specified in Eq. (16), the scaled rate of adaptation $T_c v$ follows as

$$T_c v = \frac{U_b \gamma_b}{\left(1 - \frac{\gamma_b}{\alpha_b}\right)^{\alpha_b + 1}} - \frac{U_d \gamma_d}{\left(1 + \frac{\gamma_d}{\alpha_d}\right)^{\alpha_d + 1}}, \tag{17}$$

where the quantities γ_b and γ_d denote the average scaled effect sizes of beneficial mutations, and of deleterious mutations, respectively. Note that the requirement $\gamma_b < \alpha_b$ emerges, a consequence of Eq. (2), which defines T_c. The $v = 0$ surface then follows as

$$\eta|_{v=0} = \frac{\gamma_d}{\gamma_b}\frac{\left(1 - \frac{\gamma_b}{\alpha_b}\right)^{\alpha_b + 1}}{\left(1 + \frac{\gamma_d}{\alpha_d}\right)^{\alpha_d + 1}}. \tag{18}$$

The fixation rate F can also be computed, using Eq. (1), with the result

$$F = \frac{U_b}{\left(1 - \frac{\gamma_b}{\alpha_b}\right)^{\alpha_b}} + \frac{U_d}{\left(1 + \frac{\gamma_d}{\alpha_d}\right)^{\alpha_d}}, \tag{19}$$

so that

$$\eta|_{F=U} = \frac{\left(1 - \frac{\gamma_b}{\alpha_b}\right)^{\alpha_b}}{\left(1 + \frac{\gamma_d}{\alpha_d}\right)^{\alpha_d}}\frac{\left(1 + \frac{\gamma_d}{\alpha_d}\right)^{\alpha_d} - 1}{1 - \left(1 - \frac{\gamma_b}{\alpha_b}\right)^{\alpha_b}}. \tag{20}$$

bioRxiv preprint doi: https://doi.org/10.1101/2023.07.31.551320; this version posted August 2, 2023. The copyright holder for this preprint (which was not certified by peer review) is the author/funder, who has granted bioRxiv a license to display the preprint in perpetuity. It is made available under aCC-BY 4.0 International license.

Finally, the quantity $(T_c b)^3$ simplifies to

$$(T_c b)^3 = \frac{1}{2}\left[\left(1+\frac{1}{\alpha_b}\right)\frac{T_c U_b \gamma_b^2}{\left(1-\frac{\gamma_b}{\alpha_b}\right)^{\alpha_b+2}} + \left(1+\frac{1}{\alpha_d}\right)\frac{T_c U_d \gamma_d^2}{\left(1+\frac{\gamma_d}{\alpha_d}\right)^{\alpha_d+2}}\right], \tag{21}$$

where in Eq. (21), we do not impose the constraint that $v = 0$. Below, we focus our attention on the case in which both beneficial mutations and deleterious mutations are drawn from exponential DFEs (so that $\alpha_b = \alpha_d = 1$).

A. Exponentially-distributed fitness effects

In the case $\alpha_b = \alpha_d = 1$, the $v = 0$ surface (in the space spanned by axes η, γ_b and γ_d) is described by

$$\eta|_{v=0} = \frac{\gamma_d}{\gamma_b}\left(\frac{1-\gamma_b}{1+\gamma_d}\right)^2, \tag{22}$$

and the $v = 0$ ridgeline is given by

$$\gamma_b^* = \frac{1-\sqrt{U_b/U}}{1+\sqrt{U_b/U}}, \tag{23}$$

$$\gamma_d^* = 1. \tag{24}$$

Because $p_{\text{fix}}(s) \propto e^{T_c s}$ (at least for the majority of fixed mutations, assuming validity of the MSSM approximation), the distribution of *fixed* beneficial effects is also an exponential distribution, to a good approximation (and likewise for deleterious mutations); we have $\langle s_f \rangle_b = (\Delta s_f)_b = s_b/(1 - T_c s_b)$ and $\langle s_f \rangle_d = (\Delta s_f)_d = s_d/(1 + T_c s_d)$. At the extremal point, we thus have

$$T_c \tilde{s}_b = \frac{3}{2}\left(\sqrt{1+\frac{U_d}{U_b}} - 1\right), \tag{25}$$

$$T_c \tilde{s}_d = \frac{3}{2}, \tag{26}$$

and

$$(T_c b)^3 = \frac{T_c U_d}{8}\sqrt{1+\frac{U_d}{U_b}}, \tag{27}$$

where $\tilde{s}_b \equiv \langle s_f \rangle_b + 2\,(\Delta s_f)_b$ and $\tilde{s}_d \equiv \langle s_f \rangle_d + 2\,(\Delta s_f)_d$. From Eq. (25) and Eq. (27) it follows that at the extremal point, $\tilde{s}_b \ll b$, $\tilde{s}_d \ll b$ and $T_c b \gg 1$—and thus the conditions of validity of the MSSM approximation are satisfied—if $T_c U_b \gg 1$ (under the additional assumption that $U_d \geq U_b$).

We note that in the case of exponential DFEs, Eq. (20) simplifies to

$$\eta|_{F=U} = \frac{\gamma_d}{\gamma_b}\left(\frac{1-\gamma_b}{1+\gamma_d}\right), \tag{28}$$

and $\eta|_{\alpha=0}$ can be computed using Eq. (9), with the result

$$\eta|_{\alpha=0} = \frac{\frac{1}{\gamma_d}\log(1+\gamma_d) - \frac{1}{1+\gamma_d}}{\frac{1}{1-\gamma_b} - \frac{1}{\gamma_b}\log\frac{1}{1-\gamma_b}}. \tag{29}$$

bioRxiv preprint doi: https://doi.org/10.1101/2023.07.31.551320; this version posted August 2, 2023. The copyright holder for this preprint (which was not certified by peer review) is the author/funder, who has granted bioRxiv a license to display the preprint in perpetuity. It is made available under aCC-BY 4.0 International license.

B. Arbitrary DFE

More generally, we can consider arbitrary DFEs $\rho_b(s)$ and $\rho_d(s)$ of beneficial and deleterious mutations. If we assume these DFEs have fixed shapes and consider changes in their scale, we can visualize the $v = 0$ constraint as a 2-dimensional surface within a 3-dimensional parameter space parameterized by η, γ_b and γ_d (where here γ_b denotes the average scaled effect size of a beneficial mutation, and likewise for γ_d). As in the case of single effects, a larger γ_b implies a more positive rate of adaptation, for fixed η and γ_d. Likewise, an intermediate scale of deleterious effects is maximally impactful. For a particular shape of deleterious DFE, the maximally impactful deleterious (scaled) DFE $\rho_d^*(\gamma)$, which lies on a $v = 0$ ridgeline, satisfies

$$\int \rho_d^*(\gamma) e^{-\gamma} \gamma(\gamma - 1) d\gamma = 0. \tag{30}$$

Because $e^{-\gamma}/N$ gives the fixation probability of a deleterious mutation with scaled effect γ, Eq. (30) has a simple interpretation in terms of the deleterious scaled effects which fix: on the $v = 0$ ridgeline, the *mean-squared* fixed scaled deleterious effect equates to the *mean* fixed scaled deleterious effect. This further implies that $\langle T_c s_f \rangle_d^* \leq 1$ on the $v = 0$ ridgeline (where s_f denotes a fixed mutational effect, and the expectation value averages over all deleterious fixed effects); note that this can be seen by ensuring positivity of the *variance* in fixed scaled deleterious effects, which equals $\langle T_c s_f \rangle_d^* \left(1 - \langle T_c s_f \rangle_d^*\right)$, given Eq. (30). Thus, the average fixed effect of the maximally impactful deleterious DFE, $\rho_d^*(s)$, is subject only to moderate or weak selection. The upper bound $\langle T_c s_f \rangle_d^* = 1$ is achieved for the two-effect DFE considered above, while for the two-exponential DFE we have $\langle T_c s_f \rangle_d^* = 1/2$. For the corresponding beneficial DFE, $\rho_b^*(s)$, lying on the $v = 0$ surface, a bound on $\langle T_c s_f \rangle_b^*$ is less easily established without specifying the shape of $\rho_b(s)$.

IV. EVOLUTIONARILY STABLE DFE

In the main text, we identified constraints on $U\rho(s)$ (or perhaps on $U\rho(\gamma)$, the distribution of scaled fitness effects) that yield $v = 0$ and analyzed the resulting $v = 0$ surface. In this fashion, $\rho_b(s)$ and $\rho_d(s)$ are treated as parameters which could in principle be varied independently. In some of the simplest models of genome evolution, however, $\rho_b(s)$ and $\rho_d(s)$ are not independent. For example, assuming a genome of finite length L with no epistasis, the fixation of a beneficial mutation creates an opportunity for a deleterious back-mutation of the same magnitude, and vice versa. Rice et al. (2015) consider the resulting "evolutionarily stable" DFE that is reached at long times once, for each magnitude of fitness effect $|s|$, beneficial and deleterious mutations reach a state of detailed balance, in which $\rho_b(s) p_{\text{fix}}(s) = \rho_d(s) p_{\text{fix}}(-s)$ (and consequently, the distribution of *fixed* beneficial mutations matches that of fixed deleterious mutations). A consequence of this detailed balance is that $v = 0$ for the evolutionarily stable DFE; here we discuss the application of the MSSM approximation to this state.

Given the underlying distribution $\rho_0(|s|)$ of absolute effects $|s|$, $N p_{\text{fix}}(s) \approx e^{T_c s}$ implies that

$$\rho_b(s) = \frac{\rho_0(|s|)}{1 + e^{2T_c s}}, \tag{31}$$

and

$$\rho_d(s) = \frac{\rho_0(|s|)}{1 + e^{-2T_c s}}, \tag{32}$$

so that $\int_{-\infty}^{\infty} \rho(s) s^p e^{T_c s} ds = 0$ for odd p. The average effect size $\langle s_f \rangle$ of a fixed beneficial mutation (which matches the average effect size of a fixed deleterious mutation) evaluates to

$$\langle s_f \rangle = \int_0^{\infty} \frac{\rho_0(|s|)}{e^{T_c s} + e^{-T_c s}} s ds \Big/ \left[\int_0^{\infty} \frac{\rho_0(|s|)}{e^{T_c s} + e^{-T_c s}} ds \right]. \tag{33}$$

Below, we evaluate $\langle s_f \rangle$ and related expressions for the class of stretched exponential DFEs ($\rho_0(|s|) = \frac{1}{\sigma \Gamma(1+\beta^{-1})} e^{-(s/\sigma)^\beta}$) in the $T_c \sigma \to \infty$ limit. The quantity $\langle s_f \rangle$ simplifies to

$$T_c \langle s_f \rangle \approx \frac{4C}{\pi} \approx 1.2 \tag{34}$$

bioRxiv preprint doi: https://doi.org/10.1101/2023.07.31.551320; this version posted August 2, 2023. The copyright holder for this preprint (which was not certified by peer review) is the author/funder, who has granted bioRxiv a license to display the preprint in perpetuity. It is made available under aCC-BY 4.0 International license.

where C denotes Catalan's constant. The mean-squared fixed effect (of beneficial mutations, and also of deleterious mutations) is given by

$$T_c^2 \left\langle s_f^2 \right\rangle \approx \frac{\pi^2}{4} \approx 2.5 \tag{35}$$

from which it follows that $T_c \Delta s_f \approx 1.1$. In the same limit,

$$(T_c b)^3 = \frac{\pi^3}{16} \frac{U}{\sigma \Gamma(1+\beta^{-1})} \tag{36}$$

and so $\tilde{s} \ll b$ and $T_c b \gg 1$ in the $a \to \infty$ limit if $U \gg \sigma$.

REFERENCES

Melissa, M. J., Good, B. H., Fisher, D. S., and Desai, M. M. (2022). Population genetics of polymorphism and divergence in rapidly evolving populations. *Genetics*, 221(4):iyac053.

Rice, D. P., Good, B. H., and Desai, M. M. (2015). The evolutionarily stable distribution of fitness effects. *Genetics*, 200(1):321–329.

bioRxiv preprint doi: https://doi.org/10.1101/2023.07.31.551320; this version posted August 2, 2023. The copyright holder for this preprint (which was not certified by peer review) is the author/funder, who has granted bioRxiv a license to display the preprint in perpetuity. It is made available under aCC-BY 4.0 International license.

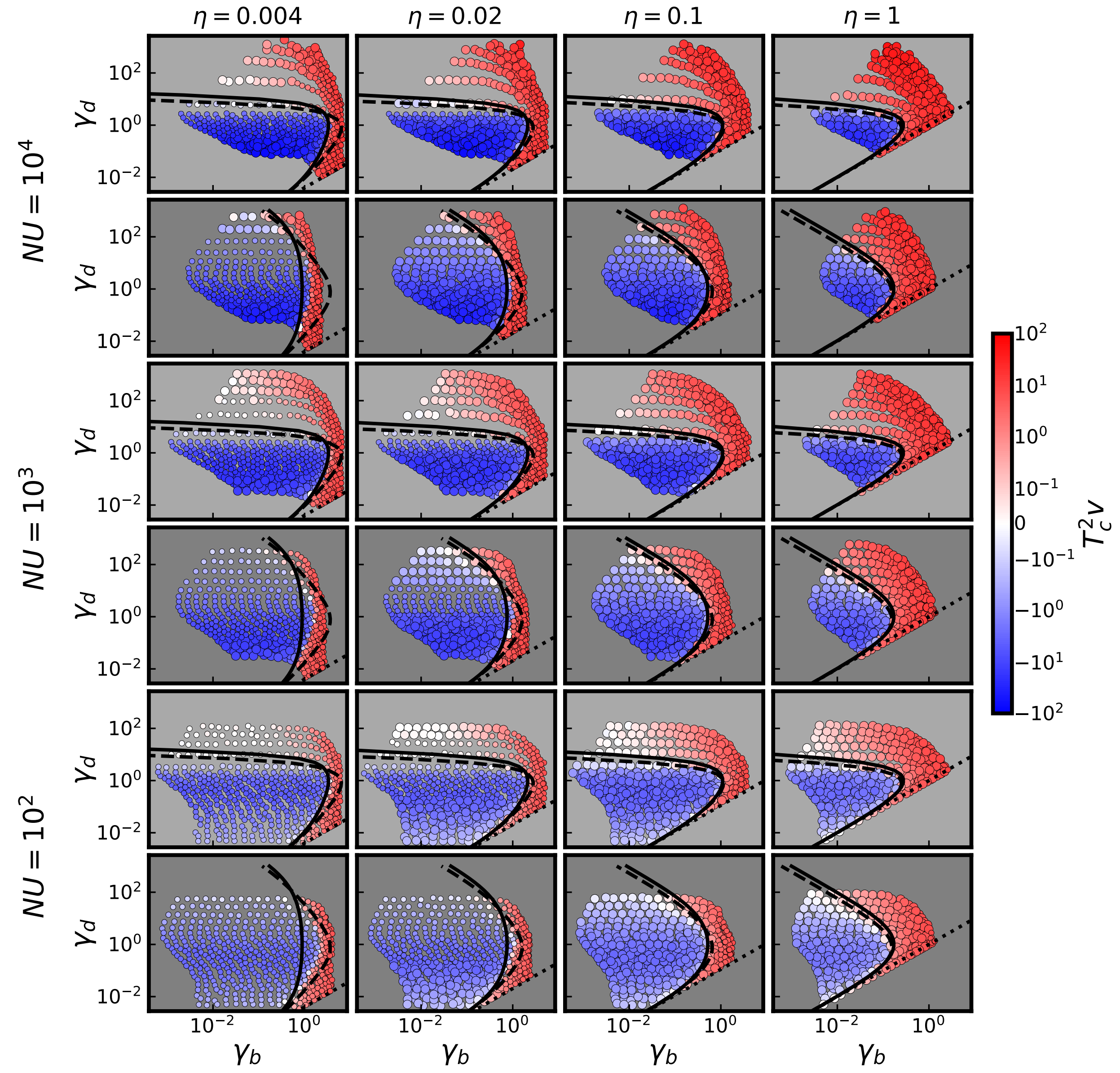

Fig. S1 Cross sections of the $v = 0$ surface in the space of scaled fitness effects. Populations are colored according to their values of $T_c^2 v$ observed in simulations. For panels colored with a dark grey background (first, third and fifth rows) populations are subject to a two-effect DFE; for panels colored with a light grey background (second, fourth and sixth rows) populations are subject to a two-exponential DFE. The parameters NU and η for simulated populations in a given panel are as denoted on the left-hand side and top of the figure, respectively. Populations with $T_c U_b < 1/2$ (which, at the $v = 0$ ridgeline, suggests the MSSM approximation may break down) are denoted by points of smaller size. Solid curves denote predictions of the MSSM approximation, dashed lines denote predictions obtained using the standard formula for fixation probabilities assuming independently evolving loci, and dotted lines are the lines $\gamma_d = \eta\gamma_b$.

bioRxiv preprint doi: https://doi.org/10.1101/2023.07.31.551320; this version posted August 2, 2023. The copyright holder for this preprint (which was not certified by peer review) is the author/funder, who has granted bioRxiv a license to display the preprint in perpetuity. It is made available under aCC-BY 4.0 International license.

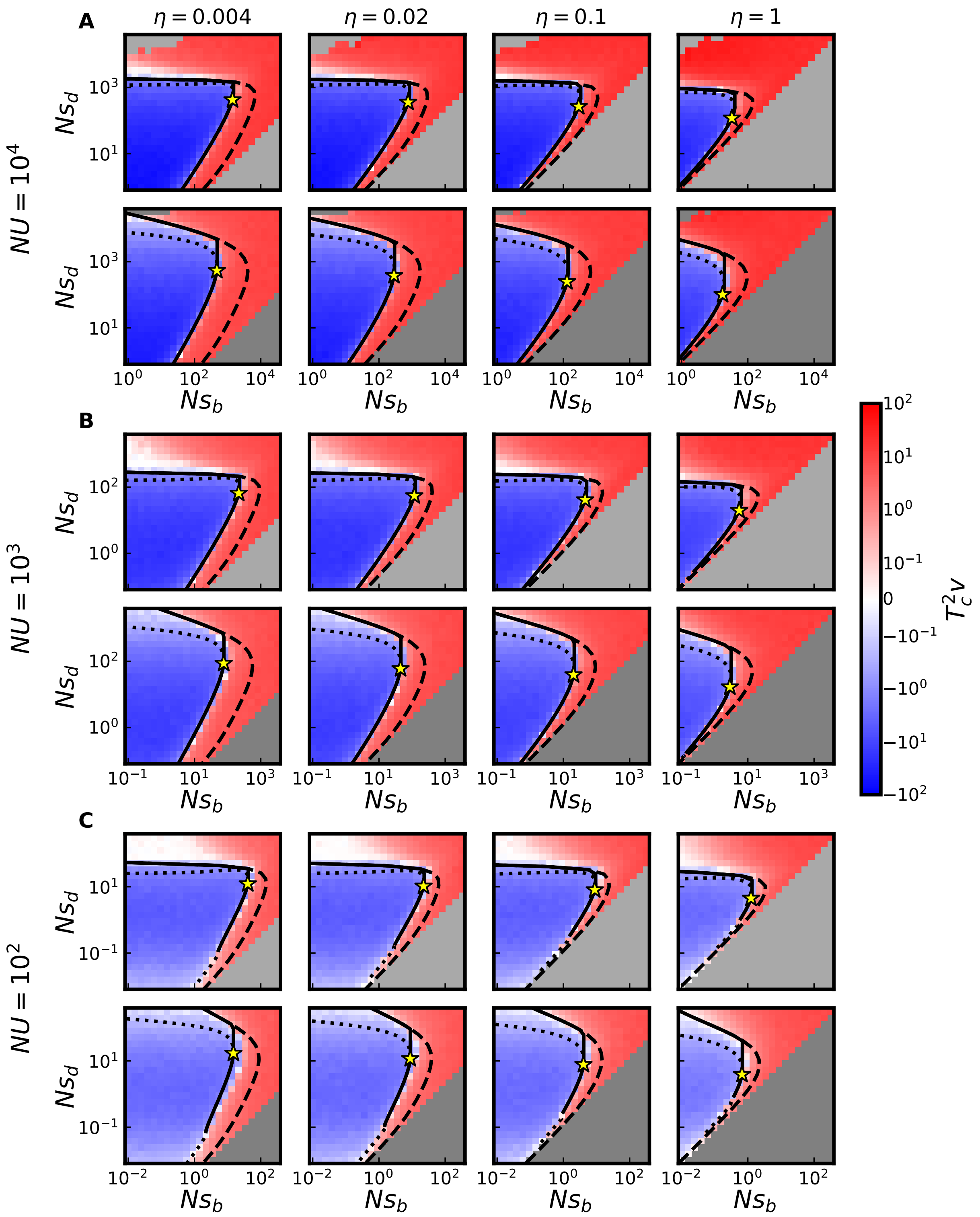

Fig. S2 Cross sections of $v = 0$ surface as obtained in simulations. Simulated populations have parameters η as depicted at the top of a given column, and $NU = 10^4$ (in A), $NU = 10^3$ (in B), or $NU = 10^2$ (in C). Populations in the first, third and fifth rows (light grey background) are subject to a two-effect DFE; populations in the second, fourth and sixth rows (dark grey background) are subject to a two-exponential DFE. Solid curves denote piecewise-defined $v = 0$ predictions given in the main text. Stars denote points at which MSSM approximation predicts $v = 0$ and $(\gamma_d, \gamma_b) = (\gamma_d^*, \gamma_b^*)$. Dashed lines are predictions obtained using our "N_e-based heuristic". Dotted lines are MSSM predictions of $v = 0$ curves obtained for parameters such that $T_c b < 1$ or $Ns_d > (Ns_d)^*$.

bioRxiv preprint doi: https://doi.org/10.1101/2023.07.31.551320; this version posted August 2, 2023. The copyright holder for this preprint (which was not certified by peer review) is the author/funder, who has granted bioRxiv a license to display the preprint in perpetuity. It is made available under aCC-BY 4.0 International license.

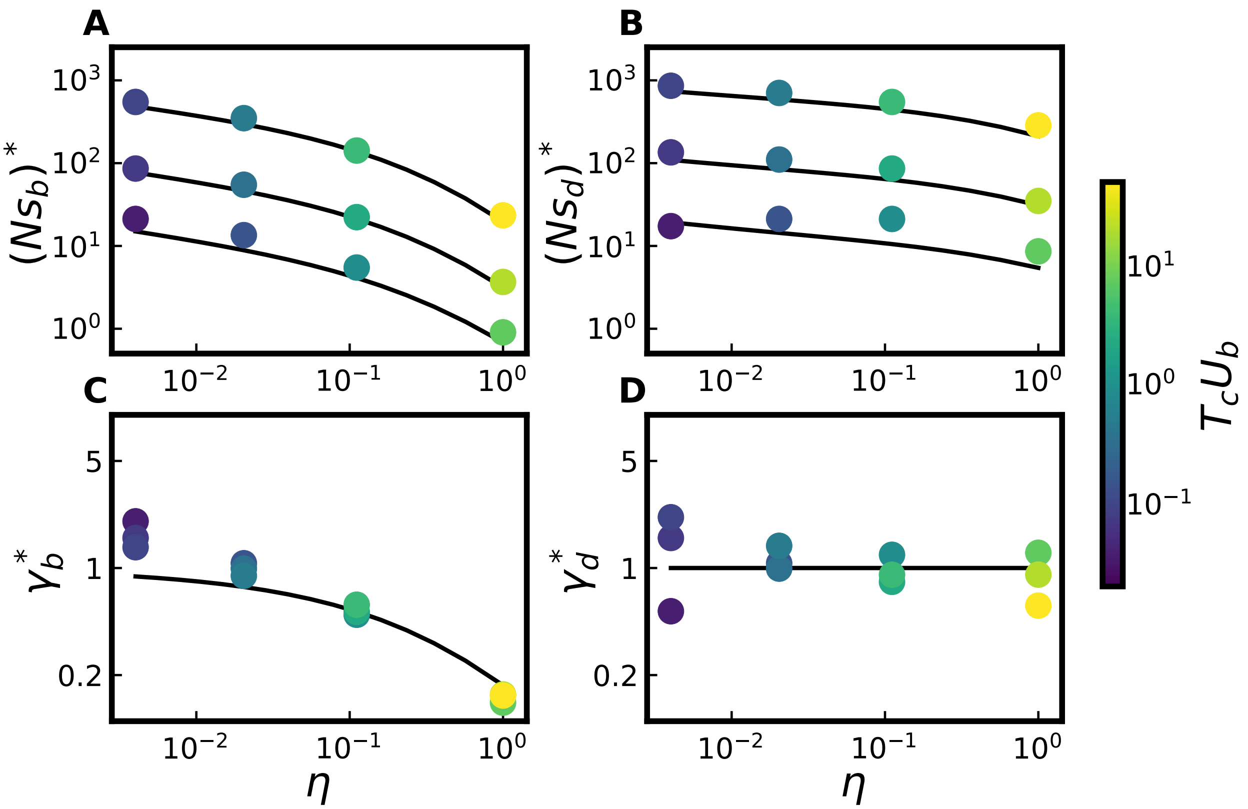

Fig. S3 Comparison between simulations and MSSM predictions for, in panels A and B, the extremal point $((Ns_b)^*, (Ns_d)^*)$ and, in panels C and D, the ridgeline point (γ_b^*, γ_d^*). Each point corresponds to a panel of Fig. S2 consisting of populations subject to a two-exponential DFE, for particular values of η and NU. In both A and B, the three solid curves are theory curves for $NU \in \{10^2, 10^3, 10^4\}$ (with the top curve corresponding to $NU = 10^4$ and the bottom curve corresponding to $NU = 10^2$). Each point is colored according to its value of T_cU_b at $(\gamma_d, \gamma_b, \eta) = (\gamma_d^*, \gamma_b^*, \eta)$.

bioRxiv preprint doi: https://doi.org/10.1101/2023.07.31.551320; this version posted August 2, 2023. The copyright holder for this preprint (which was not certified by peer review) is the author/funder, who has granted bioRxiv a license to display the preprint in perpetuity. It is made available under aCC-BY 4.0 International license.

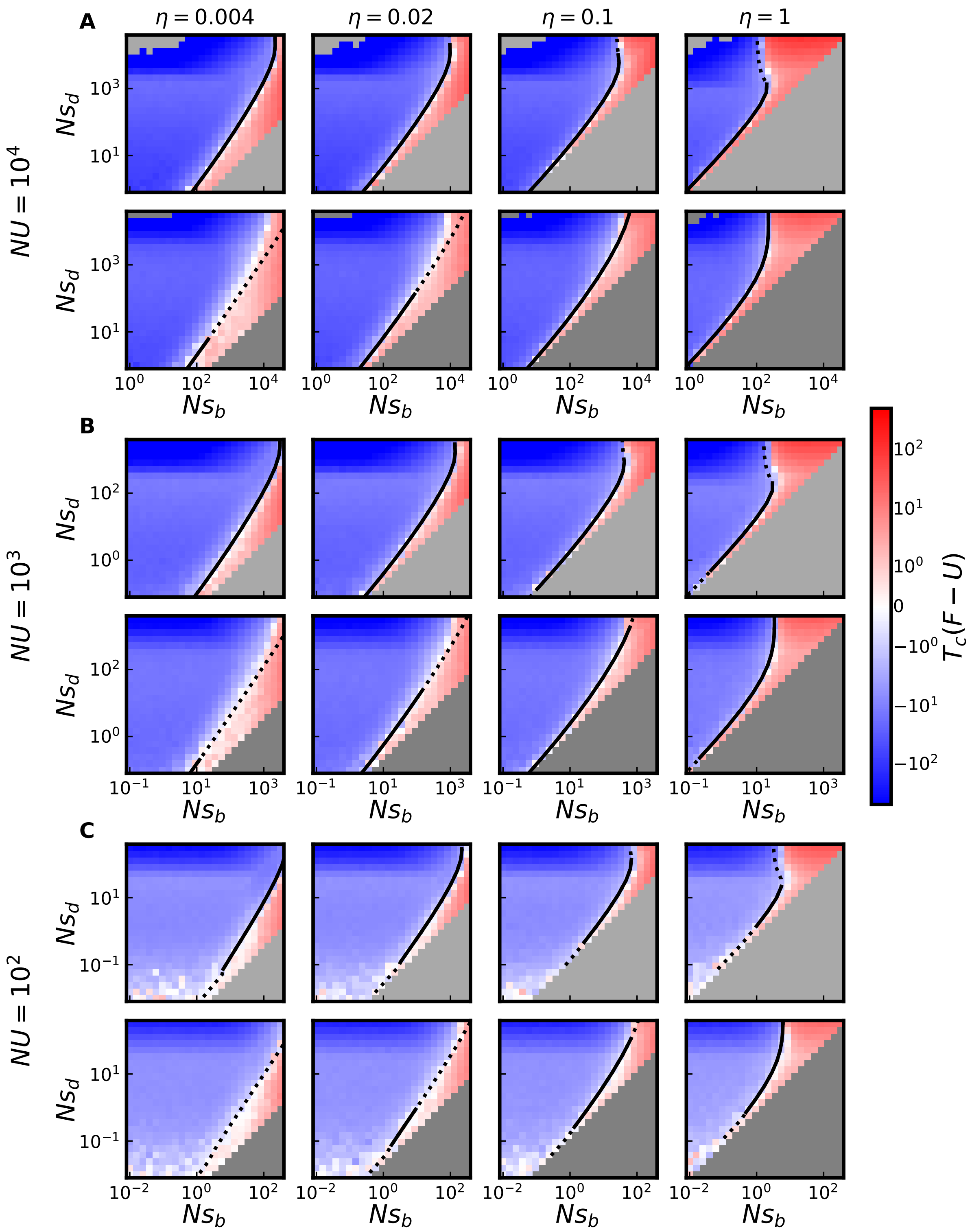

Fig. S4 Cross sections of $F = U$ surface as observed in simulations. Points are colored by values of $T_c(F - U)$ as observed in simulations. Values of NU for populations displayed in A, B and C are denoted on the left-hand side. Populations in the first, third, and fifth row are subject to two-effect DFEs; populations in the second, fourth, and sixth rows are subject to two-exponential DFEs. Solid curves denote MSSM predictions for $F = U$ surface, with dotted lines denoting these predictions beyond the regime of validity of the MSSM approximation.

bioRxiv preprint doi: https://doi.org/10.1101/2023.07.31.551320; this version posted August 2, 2023. The copyright holder for this preprint (which was not certified by peer review) is the author/funder, who has granted bioRxiv a license to display the preprint in perpetuity. It is made available under aCC-BY 4.0 International license.

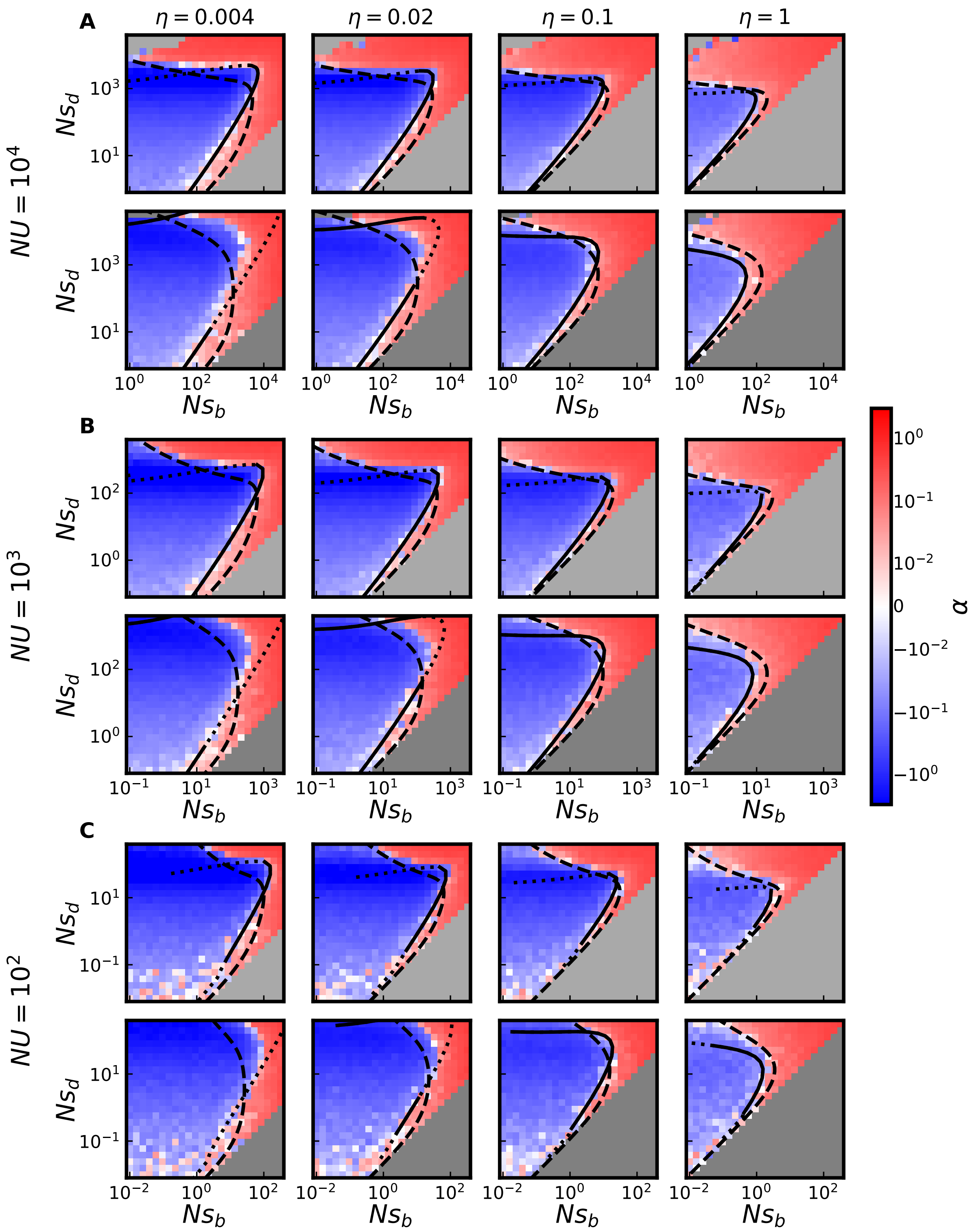

Fig. S5 Cross sections of $\alpha = 0$ surface as observed in simulations. Points are colored by values of α as observed in simulations. Values of NU for populations displayed in A, B and C are denoted on the left-hand side. Populations in the first, third, and fifth row are subject to two-effect DFEs; populations in the second, fourth, and sixth rows are subject to two-exponential DFEs. Solid curves denote MSSM predictions for $\alpha = 0$ surface, with dotted lines denoting these predictions beyond the regime of validity of the MSSM approximation. Dashed lines denote predictions obtained by using our "N_e-based heuristic" to compute T_c instead of the MSSM approximation.
